# Overview of current targeted therapy in gallbladder cancer

**DOI:** 10.1038/s41392-020-00324-2

**Published:** 2020-10-07

**Authors:** Xiaoling Song, Yunping Hu, Yongsheng Li, Rong Shao, Fatao Liu, Yingbin Liu

**Affiliations:** 1 Department of General Surgery and Laboratory of General Surgery, Xinhua Hospital Affiliated to Shanghai Jiao Tong University School of Medicine, 1665 Kongjiang Road, 200092 Shanghai, China; 2 Shanghai Key Laboratory of Biliary Tract Disease Research, 1665 Kongjiang Road, 200092 Shanghai, China; 3 State Key Laboratory of Oncogenes and Related Genes, Shanghai Cancer Institute, School of Medicine, Shanghai Jiao Tong University, 200127 Shanghai, China; 4 Department of Biliary-Pancreatic Surgery, Renji Hospital, School of Medicine, Shanghai Jiao Tong University, 200127 Shanghai, China; 5 Department of Pharmacology, Shanghai Jiao Tong University School of Medicine, 200025 Shanghai, China

**Keywords:** Gastrointestinal cancer, Drug development

## Abstract

Gallbladder cancer (GBC) is rare, but is the most malignant type of biliary tract tumor. Unfortunately, only a small population of cancer patients is acceptable for the surgical resection, the current effective regimen; thus, the high mortality rate has been static for decades. To substantially circumvent the stagnant scenario, a number of therapeutic approaches owing to the creation of advanced technologic measures (e.g., next-generation sequencing, transcriptomics, proteomics) have been intensively innovated, which include targeted therapy, immunotherapy, and nanoparticle-based delivery systems. In the current review, we primarily focus on the targeted therapy capable of specifically inhibiting individual key molecules that govern aberrant signaling cascades in GBC. Global clinical trials of targeted therapy in GBC are updated and may offer great value for novel pathologic and therapeutic insights of this deadly disease, ultimately improving the efficacy of treatment.

## Introduction

Gallbladder cancer (GBC) is the most common cancer of the biliary tract system and ranked as the top six in general gastrointestinal tract neoplasms worldwide.^1–3^ While the incidence rate of GBC varies widely, it has a unique distribution pattern in some regions, where Chile, India, some other Asian countries, Eastern European, and Latin American countries have reported more cases than the rest of the world every year.^4–6^ This geographical distribution signature probably ascribed to the difference of genetic susceptibility contributes to an important risk factor of GBC. The other factors, which associated with chronic inflammation and disease pathogenesis, such as hepatobiliary stones, liver flukes, and Clostridium frequently observed in these areas, also constitute the other high-risk factors of bile tract cancer (BTC) including GBC.^7^ In addition to these regionally limited factors, plenty of ubiquitous risk factors have been documented globally and taken into account seriously, which include gallstone, gender, age, obesity, reproductive factors, race, primary sclerosis cholangitis, gallbladder polyps, congenital biliary cysts, typhoid, *Helicobacter pylori* infection, alcohol intake, smoking, fatty liver disease, unhealthy diet, and environmental exposure to specific chemicals.^8–10^ As a result, the early protection from carcinogenesis has been enforced in clinical practice and the occurrence of this disease only accounts for 1.2% of all cancers diagnosed in the world.

Currently, radical resection is the most effective strategy to potentially cure GBC. Unfortunately, the population falling into this operational course is largely limited, as a large number of patients (>70–90%) can only accept non-surgical treatment. Such unfavorable outcome is because of the atypical clinical symptoms at earlier stages, contrary to the noticeable symptoms that emerge in most cases with advanced disease.^11,12^ The non-surgical therapies engaged in patients were primarily composed of chemotherapy and radiotherapy. However, over the past decades, additional therapeutic strategies have been continuously renovated, given rapid discoveries of the advanced technology, including next-generation sequencing (NGS), whole-exome sequencing (WES), RNA-sequencing (RNAseq), and single-cell isolation, as well as characterization that have fundamentally opened a novel view enabled to globally identify genetic and epigenetic features and key molecules as potential therapeutic targets. In particular, specific target treatment, immune therapy, vaccine therapy, biotherapy, and nanoparticles have been intensively developed in the preclinical and clinical trials. In the present review, we will focus on the targeted regimen and immune therapy as these treatments have recently received considerable attention with the hope of improving quality of life and overall survival (OS) of GBC patients in the clinic.

## Chemotherapy and radiotherapy of GBC

Chemotherapy involves agents used for non-specific inhibition of tumor cell proliferation usually via blockade of DNA synthesis, which has been extensively engaged in the treatment of a variety of cancers. National Comprehensive Cancer Network has provided two options for GBC treatment: single-agent therapy, which is fluoropyrimidine or gemcitabine-based treatment, and multiagents regimen, which includes oxaliplatin, cisplatin, and capecitabine.^13–15^ Although there are limited data to define a standard regimen or definitive benefit, the combined therapy regimens of FOLFOX (5-fluorouracil and oxaliplatin),^16^ CAPOX (capecitabine and oxaliplatin),^17,18^ GC (gemcitabine and cisplatin),^19^ and Gemox (gemcitabine and oxaliplatin)^20^ still remain the mainstream chemotherapy programs in clinical trials. It is noticeable that some clinical trials have shown that combination chemotherapy with fluorouracil, leucovorin, irinotecan, and oxaliplatin (FOLFIRINOX) yield promising results in patients with BTC.^21,22^ However, none of the single program has been widely offered, since unexpected responses were deliberately taken into account, such as systemic toxicity, insufficient drug responses, and drug resistance.^23^ Therefore, there are currently a large number of preclinical and clinical researches that are adding significant efforts in order to define the overall benefit of drug treatment even in the presence of adversary responses, which can be otherwise governed at a minimal level. For example, transplantation of freshly resected patient tissue of GBC in mice as a mini patient-derived xenograft (mini-PDX) model to examine individual drug sensitivity and select more effective drugs from combined agent trials for the guidance of clinic drug-selective treatment in the patient. They tested gemcitabine, oxaliplatin, 5‐fluorouracil, nanoparticle albumin‐bound nab‐paclitaxel, and irinotecan after surgery, and found that patients in the PDX-guided chemotherapy group had significantly longer median OS (mOS; 18.6 months; 95% confidence interval (CI) 15.9–21.3 months) and disease-free survival (DFS 17.6 months; 95% CI 14.5–20.6 months) than patients in the conventional random drug-selective chemotherapy group with mOS (13.9 months; 95% CI 11.7–16.2 months) (*P* = 0.030; hazard ratio (HR) 3.18; 95% CI 1.47–6.91) and DFS (12.0 months; 95% CI 9.7–14.4 months) (*P* = 0.014; HR 3.37; 95% CI 1.67–6.79). Thus, mini-PDX may devise the regimes of chemotherapy to improve the outcomes; in particular, providing an optimal opportunity for personalized medicine.^24,25^

In addition to intervention of most chemotherapeutic agents on DNA synthesis, over the past decade, there has been growing research work focusing on RNA molecules that controls oncogene or tumor suppressor gene expression. It is emerging that microRNAs (miRNAs) and long non-coding RNAs (lncRNAs) act as indispensable factors to coordinately manipulate multiple gene expression underlying carcinogenesis, in sharp contrast to the traditional paradigm that non-coding RNAs are noisy and non-functional in the regulation of gene expression. Multiple laboratories including ours have reported that miRNAs and lncRNAs mediate the proliferation, invasion, and chemotherapy resistance of GBC, and serve as new therapeutic targets for the treatment of advanced GBC, including miR-125b-5p,^26^ miR-122,^27^ miR-223,^28^ miR-31,^29,30^ miR-30a-5p,^31^ lncRNA-HGBC,^32^ lncRNA-PAGBC,^33^ lncRNA PVT1,^34^ and lncRNA GBCDRlnc1.^35^ Concomitantly, advanced nanotechnology employing various modified materials that offer optimal cargos to efficiently deliver RNA molecules has also given rise to promising benefit in the chemotherapy of GBC. Cai and co-workers^36,37^ found that nanomaterial-induced photothermal therapy in combination with chemotherapy and chloroquine inhibited GBC cell proliferation. It is worthwhile monitoring the efficacy of this novel delivery system with RNA molecules as an alternative therapy for GBC in future.

Albeit the benefit of adjuvant radiotherapy (ART) is evident in many other types of cancers, it remains unclear in GBC, since there is a lack of strong evidence of improved endpoints. Up to date, no standard regimens are available for the selection of ART; thus, combination therapy of ART with chemotherapy is usually engaged in the clinic. Some of the clinical settings have yielded encouraging results.^38^ For instance, a phase II study led by the Southwest Oncology Group (SWOG) investigated the clinical outcomes of extrahepatic cholangiocarcinoma and GBC treated with adjuvant chemotherapy GC followed by chemoradiation (combined capecitabine and radiotherapy) in a single-arm study. The 2-year OS was 65% for all patients (67% for R0 and 60% for R1), the mOS time was 35 months, and only 14 patients had local recurrence, demonstrating the feasibility and benefit of adjuvant chemotherapy followed by chemoradiation therapy for GBC.^39^ Thus, the combination of radiotherapy is well tolerated, and has a considerable effect; however, it still needs to be confirmed by large-scale, multicenter randomized case–control phase III clinical trials.

## Targeted therapy of GBC

**Fig. 1:**
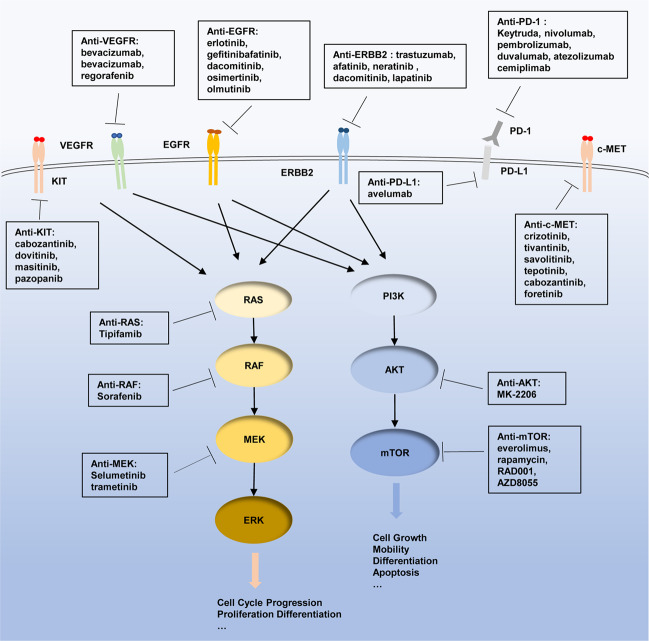
Overview of GBC targeted sites and agents. Boxes highlight drugs undergoing clinical investigation as reviewed, with arrows indicating pathway/target activation and blocked lines indicating pathway/target inhibition. c-MET mesenchymal–epithelial transition factor, VEGF vascular endothelial growth factor, VEGFR vascular endothelial growth factor receptor, EGFR epidermal growth factor receptor, ERBB2 human epidermal growth factor 2, PD-1 programmed death-1, PD-L1 programmed death ligand 1, PI3K phosphoinositide 3-kinase, AKT protein kinase B, also known as PKB, mTOR mammalian target of rapamycin, MEK mitogen-activated protein kinase, ERK extracellular signal-regulated kinase

Targeted therapy for cancers was initially developed in 1988 based on the concept of specific chemicals that was able to eliminate some microorganisms in the early 1900.^40^ Since then the efficacy of targeted therapy has been extensively investigated in multiple cancers to specifically block a number of molecular targets that are keenly associated with tumor cell proliferation, differentiation, migration, cancer stemness, vascular angiogenesis, and antitumor immune responses.^41,42^ A great volume of drugs for targeted therapy have been created, which mainly consist of small molecules and immunized antibodies. The small molecules with molecular weight <900 Da can be readily transported into cells to inactivate specific proteins or enzymes, thus inhibiting tumor cell growth,^43^ while therapeutic antibodies specifically bind to cell membrane receptors or their ligands to regulate cell proliferation or apoptosis.^44^ Some drugs were developed to target extracellular molecules that mediate angiogenesis or immune reaction in the tumor microenvironment, resulting in inhibition of tumor growth, angiogenesis, and metastasis.^45,46^ With regards to recently intense clinical research on varied specific agents that intervene intracellular signaling pathways dysfunctional in GBC (Table 1), here we updated these targeted therapies on individual signaling pathways, including human epidermal growth factor receptor 2 (HER2), growth factor receptor tyrosine kinases (RTKs) (EGFR, vascular endothelial growth factor receptor (VEGFR)), programmed death receptor 1 (PD-1)/programmed death ligand 1 (PD-L1), TP53, KIT, CDKN2A/B, phosphatidylinositol 3-kinase (PI3K)/AKT/mammalian target of rapamycin (mTOR), and RAS/BRAF/MEK/MAK (Fig. 1).

**Table 1:** Ongoing clinical trials evaluating gallbladder cancer (GBC)

Drug investigated	Molecular target	Target population	Phase	Clinical trail ID	Locations
Sorafenib	Multitargeted TKI	GBC	2	NCT00238212	USA
Sorafenib	Multitargeted TKI	Extrahepatic bile duct cancer, GBC	2	NCT00919061	USA
Sorafenib	Multitargeted TKI	BTC	1 and 2	NCT00955721	USA
Sorafenib	Multitargeted TKI	GBC	3	NCT01053390	China
KBP-5209	Multitargeted TKI	Solid tumors	1	NCT02442414	USA
Erlotinib	EGFR	Solid tumors	1	NCT00397384	USA
Bevacizumab	EGFR, VEGFR	Upper gastrointestinal cancers	2	NCT00350753	Denmark
Bevacizumab	EGFR, VEGFR	BTC	2	NCT00356889	USA
Bevacizumab	EGFR, VEGFR	BTC	2	NCT00361231	USA
Bevacizumab	EGFR, VEGFR	BTC	2	NCT01007552	USA
Afatinib	EGFR, HER2	GBC	2	NCT04183712	China
Apatinib	EGFR, HER2	GBC	2	NCT03702491	China
Lapatinib	HER2	BTC	2	NCT00101036	USA
Lapatinib	HER2	BTC	2	NCT00107536	USA
Trastuzumab, R115777	HER2	Solid tumors	1	NCT00005842	USA
Trastuzumab	HER2	Advanced or metastatic GBC	2	NCT00478140	USA
Trastuzumab, IL-12	HER2, IL-12	Solid tumors	1	NCT00004074	USA
IL-2	HER2	Solid tumors	1	NCT02662348	China
Cediranib	VEGFR	BTC	2	NCT01229111	USA
Ramucirumab	VEGFR2	BTC	2	NCT02520141	USA
Ramucirumab, merestinib	VEGFR2, c-MET	BTC	2	NCT02711553	USA
Pazopanib	VEGFR1, VEGFR2, VEGFR3, PDGFRβ, c-Kit, FGFR1, c-Fms	BTC	2	NCT01855724	Greece
Vandetanib	VEGFR2–3, EGFR, RET	Advanced BTC	2	NCT00753675	Italy
Regorafenib	VEGFR1–3, PDGFRβ, KIT, RET Raf-1	BTC	2	NCT02115542	USA
Regorafenib	VEGFR1–3, PDGFRβ, KIT, RET Raf-2	BTC	2	NCT02053376	USA
Panitumumab	Kras, BRAF	BTC	2	NCT01308840	USA
Selumetinib	MEK	BTC	1	NCT01242605	United Kingdom
Selumetinib	MEK	BTC	2	NCT02151084	Canada
Atezolizumab	MEK	BTC	2	NCT03201458	USA
Trametinib	MEK	BTC or GBC	2	NCT02042443	USA
Trametinib	MEK	BTC	2	NCT01943864	Japan
ARRY-438162	MEK	Solid tumors	1	NCT00959127	USA
GSK1120212	MEK	Solid tumors	1	NCT01324258	Japan
MEK162	MEK	BTC	1	NCT02105350	USA
MEK162	MEK	BTC	1 and 2	NCT01828034	USA
MEK162	MEK	BTC	1 and 2	NCT02773459	Korea
Everolimus	mTOR	Solid tumors	1	NCT00949949	USA
Nivolumab	PD-1	BTC	2	NCT02829918	USA
Nivolumab	PD-1	BTC	2	NCT03101566	USA
Pembrolizumab	PD-1	BTC	2	NCT03260712	Spain
Pembrolizumab	PD-1	BTC	2	NCT03111732	USA
Pembrolizumab	PD-1	BTC	3	NCT04003636	USA
M7824	PD-1	BTC	2	NCT03833661	USA
Toripalimab + lenvatinib	PD-1	BTC	2	NCT04211168	China
Nivolumab, ipilimumab	PD-1, CTLA-4	Solid tumors	2	NCT02834013	USA
STI-3031	PD-L1	BTC	2	NCT03999658	USA
Avelumab	PD-L1	Solid tumors	1 and 2	NCT04068194	USA
Durvalumab	PD-L1	BTC	2	NCT04308174	Korea
Durvalumab/tremelimumab	PD-L1, CTLA-4	BTC	2	NCT03473574	Germany
Intrafusp alfa	PD-L1, TGF-β	BTC	2 and 3	NCT04066491	USA
Selumetinib	AKT	BTC	2	NCT01859182	USA
MK-2206	AKT	BTC	2	NCT01425879	USA
IL-12	IL-12	Solid tumors	1	NCT00003046	USA
IL-12	IL-12	Solid tumors	1	NCT00003439	USA
Guadecitabine	DNMT	Advanced liver, pancreatic, BTC, GBC	1	NCT03257761	USA
CEA RNA-pulsed DC cancer vaccine	CEA	Solid tumors	1	NCT00004604	USA
EphB4-HSA fusion protein	EphB4, HSA	Solid tumors	1	NCT02495896	USA
ADH-1	N-cadherin	Solid tumors	1	NCT01825603	USA
CPI-613	PDH, α-KGDH	BTC	1 and 2	NCT04203160	USA
Glivec	ABL, KIT, PDGFR	BTC	2	NCT01153750	Germany
DKN-01	DKK1	BTC	1	NCT02375880	USA
PSMA/PRAME	T cells	Solid tumors	1	NCT00423254	USA
Merestinib	MET	Solid tumors	1	NCT03027284	Japan
FT-2102	IDH1	Solid tumors	1 and 2	NCT03684811	USA
Entinostat	HDAC	Solid tumors	1	NCT00020579	USA
CGX1321	PORCN	Solid tumors	1	NCT03507998	China
Ceralasertib	PARP	Solid tumors	2	NCT03878095	USA

### HER2

HER2, also known as ErbB2, is a member of HER family, which includes HER1, HER2, HER3, and HER4, also called ErbB1 (EGFR), ErbB2, ErbB3, and ErbB4, respectively.^47,48^ HERs are cell-surface receptors that harbor a transmembrane tyrosine kinase domain capable of activating multiple downstream signal pathways upon binding with epidermal growth factor (EGF).^49^ HER2 has been well appreciated to play a crucial role in cancer biology and is an essential functional partner for the other family member receptor binding orchestrating a heterodimer, while HER2 itself forms a homodimer as cognate ligand binding.^50^ HER2/HER3 interaction leads to PI3K/Akt phosphorylation and downstream signaling activation that mediates cell polarity, cell adhesion, and cell cycles.^51,52^ HER2 can also trigger mitogen-activated protein kinase (MAPK) signaling.^53^ Many elegant mechanistic studies on these signaling pathways reported previously were not elucidated here.

Abnormality of HER2 with gene overexpression and/or activated mutations has been reported in multiple cancers, such as breast cancer, colon cancer, lung cancer, stomach cancer, and BTC.^50,54,55^

**Fig. 2:**
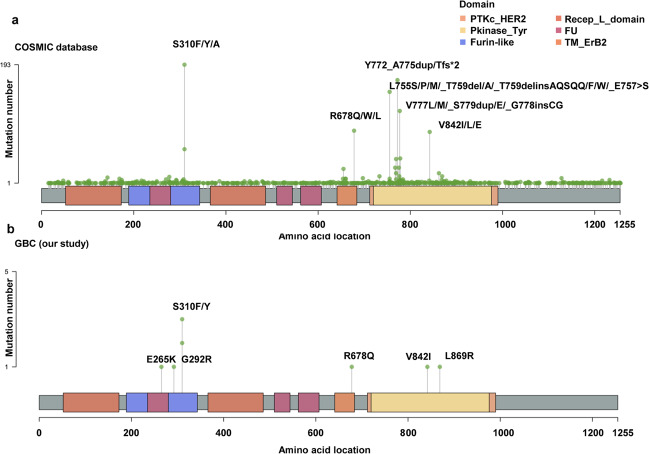
Summary of HER2 mutations in the COSMIC database (**a**) and mutation information of HER2 in our previous two studies (**b**)

Kiguchi et al.^56^ first reported that overexpression of HER2 in the basal layer of biliary tract epithelium in transgenic mice led to the development of gallbladder adenocarcinoma at the age of 3 months. In human GBC, HER overexpression was found between 9.8 and 12.8%.^54,57^ Since 2014, we have added notable effort to gain GBC WES and identify potential mutations of ERBB family. In Fig. 2b, we summarized the detailed mutation information of HER2 discovered in our previous two studies. Frequency of mutations of HER2 and HER3 was found to be 9.8% and 11.8%, respectively, which accounted for ErbB signaling pathway activations in GBC.^58^ Supporting the evidence, these activated mutations of HER2/3 in GBC cell lines resulted in a significant increase in cell proliferation and tumor development in animals,^59^ underscoring the essential role of HER2/3 mutations in the development of GBC. In concert with our findings, Sebastian and co-workers^60^ also recently demonstrated the high alteration frequency (12.6%) of HER2 in GBC and the S310 of HER2 as a hot-spot mutation.

There was growing research evidence reported from preclinical and clinical therapeutic trials in GBC. Ah-Rong and co-workers^61^ found that HER2^+^ SNU-2670 and SNU-2773 GBC cell lines were more sensitive to trastuzumab, dacomitinib, and afatinib than HER2^−^ BTC cell lines. In addition, in the mouse xenograft model of SNU-2670, trastuzumab as a monotherapy and in combination with gemcitabine demonstrated a stronger antitumor effect and greater cell apoptosis than gemcitabine treatment. Iyer et al.^62^ discovered that treatment of GBC cells isolated from patients with ErbB2-specific short hairpin RNA (shRNA), EGFR-specific shRNA, or afatinib can inhibit the invasiveness of GBC cells. Interestingly, Wang et al.^63^ showed that gemcitabine/5-fluorouracil increased the expressions of total and phosphorylated forms of HER2 in GBC cells, thus enhancing the cytotoxicity of trastuzumab, suggesting that sequential therapy with gemcitabine/5-fluorouracil followed by trastuzumab perhaps devises a novel and promising therapeutic strategy aiming at HER2^−^ GBCs that are currently short of targeted drugs.

Inagaki et al.^64^ reported that a GBC case harboring HER2 mutation on the primary and metastatic site underwent HER2-targeted treatment with lapatinib and capecitabin. After two cycles of treatment, contrast computed tomography imaging showed a decrease in the size of tumor emboli and hepatic lesions. Likewise, Prieto et al.^65^ demonstrated favorable benefit of 5-year survival without recurrence after treatment with chemotherapy and trastuzumab on a stage IV GBC patient who presented progression during first-line chemotherapy treatment following radical savage surgery. It is emerging that the insufficiency of GBC cohorts enrolled in the studies is the primary obstacle for the clinical trial practice. In 2009, Ramanathan et al.^66^ reported a phase II study with lapatinib on patients with advanced biliary tree and hepatocellular cancer previously treated with no more than one line of chemotherapy. Of 17 BTC patients, 5 patients with GBC did not achieve any improvement with the treatment. In 2019, Harding et al.^67^ operated another phase II SUMMIT “basket” trial, a multi-histology, open-label, phase II “basket” study for patients who harbored somatic HER2 mutations and received neratinib (ClinicalTrial.gov NCT01953926; EudraCT 2013-002872-42). A total of 19 BTC patients containing 8 GBCs were recruited for a single-arm trial with neratinib monotherapy at 240 mg oral daily. Calculating all BTC patients as a whole, the confirmed objective response rate (ORR) was 10.5% (95% CI 1.3–33.1) and clinical benefit rate was 31.6% (95% CI 12.6–56.6), including two confirmed partial response (PR) and four patients achieving stable disease. mPFS was 1.8 months (95% CI 1.0–3.7). These data indicate that HER2-directed targeted therapy represents a novel and tolerable treatment approach for advanced BTCs that express somatic HER2 mutations.

We have recently begun a clinical trial named “A Multicenter, Open-label, Randomized, Controlled Study of Target Therapy Based on Tumor Molecular Profiling With GEMOX in Resectable GBC Patients Monitored by ctDNA.” (ClinicalTrial.gov NCT04183712). A total of 54 GBC participants will be enrolled from multiple hospitals and the feasibility, efficacy, and safety of target therapy with afatinib will be evaluated and the outcomes of this study will be reported in 2 years.

### VEGF/VEGFR

VEGF/VEGFR axis plays a key role in both physiological and pathological vascularization in diseases such as tumor angiogenesis.^68–71^ There are five distinct VEGF family members in a mammal: VEGF-A (also referred to as VEGF), VEGF-B, VEGF-C, VEGF-D, and placenta growth factor (PLGF). VEGFR family consists of RTK member, including VEGFR1, VEGFR2, and VEGFR3, as well as the non-tyrosine kinase co-receptors neuropilin-1 (NP-1) and NP-2. The tyrosine kinase receptors are composed of an extracellular ligand-binding region with distinct binding affinities for individual VEGF ligands, seven immunoglobulin-like loop domains, and a cytoplasmic catalytic domain.^72,73^ VEGF-A, VEGF-B, and PLGF are mainly involved in angiogenesis, while VEGF-C and VEGF-D regulate lymphangiogenesis. VEGF-A and VEGF-B display the rigorous ability to interact with VEGFR1 and VEGFR2 expressed on vascular endothelial cells and vascular smooth muscle cells.^74^ VEGF-C and VEGF-D have high affinity to bind to VEGFR3 expressed on lymphoendothelium, stimulating lymphangiogenesis.^75^

VEGFR1 was expressed on many types of cells, including endothelial cells, epithelial cells, inflammatory cells, and cancer cells. Interestingly, VEGFR1 does not seem to regulate endothelial cell migration or proliferation;^76,77^ instead, VEGFR1 regulates epithelial cell differentiation and migration.^78^ In endothelial cells, VEGFR1 is recognized as a decoy to regulate free VEGF-A as it binds to and activates VEGFR2.^79^ The binding of VEGF to VEGFR2 leads to phosphorylation of VEGFR2 at Tyr951 and Tyr1175, in which phosphorylated Tyr951 regulates vascular permeability mediated by SRC tyrosine kinase activation,^80^ whereas phosphorylated Tyr1175 of VEGFR2 recruits phospholipase Cγ (PLC-γ) and activates downstream of both MAPK cascade and PI3K/AKT pathway, stimulating endothelial cell proliferation and survival.^72,81^ Activated VEGFR3 induces the RAS/MAPK/ERK pathway and the PI3K-AKT/PKB pathway, leading to increased differentiation, migration, proliferation, and survival of lymphatic endothelial cells.^82,83^ Intriguingly, VEGFR3 was also documented to fenestrate VEGF-A/VEGFR2 signaling, participating in angiogenesis.^84^

In addition to vast research evidence revealing the regulatory role of the VEGF–VEGFR axis, the cooperative or independent impacts of the axis-associated molecules in GBC have also been concomitantly explored. For example, HIF‑1α was found to promote tumor cell migration via upregulation of VEGF-A in GBC; this effect was inhibited by metformin.^85^ Tumor necrosis factor-α (TNF-α)-induced ERK1/2-AP-1 pathway-dependent transcriptional activation of VEGF-D, leading to lymphangiogenesis and lymphatic metastasis of GBC.^86^ Likewise, receptor-interacting protein 1, a multifunctional protein in the TNF-α signaling pathway, was highly expressed in GBC and promoted lymphangiogenesis and lymph node metastasis via nuclear factor-κB-mediated transcriptional activation of VEGF-C.^87^ Dual-specificity MAP kinase phosphatase1 (DUSP1/MKP1) suppressed VEGF expression, abrogating angiogenesis in GBC mouse model.^88^ In addition, overexpression of miR-1 in GBC cells inhibited VEGF-A mRNA expression.^89^

It is well appreciated that microvessel density correlated with cancer progression, metastasis, and prognosis in GBC^90^ and VEGF-A was overexpressed to serve as an independent prognostic factor of survival in GBC.^91^ Recently, Xu et al.^92^ found that VEGF was notably elevated in the serum of patients with GBC and VEGF promoted angiogenesis, cell proliferation, and invasion, but inhibited apoptosis in GBC cells. Inconsistent with the data, Zhang et al.^93^ reported that the expression of estrogen receptor 1 (ER1) or VEGF-A alone was not correlated with OS of GBC patients. However, combined high expression of VEGF-A with ER1 predicted poor prognosis for GBC patients, suggesting that VEGF-A combined with hormone receptor ER may provide a biomarker for GBC prognosis. Resembling tumor angiogenesis, lymphoangiogenesis was known to play a central role in GBC metastasis. In a case–control study with 50 patients of GBC, 10 samples of normal gallbladder tissues and 19 samples of chronic cholecystitis, VEGF-C and -D were overexpressed in GBC tissues relative to normal or inflammation tissues.^94^ Strongly supporting this evidence, a number of independent studies have found that serum and tumor VEGF-C levels were increased in patients with GBC compared with healthy donors. In addition, the elevated serum VEGF-C was positively correlated with decreased OS and increased lymph node metastasis.^95,96^ In a large cohort study with 195 GBC patients and 300 healthy serum samples, polymorphisms of c.*237C > T and g.43737830A > G of VEGF gene were associated with the disease development, indicating that VEGF polymorphisms may offer a valuable marker to predict the susceptibility of carcinogenesis.^97^ In line with clinic trials, blocking VEGF-C by short interfering RNA or a neutralizing antibody in cultured GBC cells inhibited tumor cell proliferation and invasion. Indeed, using orthotopic xenograft models, Lin et al.^98^ found that inhibition of VEGF-D led to the suppression of lymphangiogenesis and lymphatic metastasis.

As a variety of anti-angiogenic inhibitors, including antibodies and small molecules, were frequently engaged in multiple cancer patients, these inhibitors have also been increasingly employed in the clinical practice for GBC patients. A multicentric phase II study of VEGF antibody bevacizumab (NCT00361231) in combination with gemcitabine and oxaliplatin in advanced BTC with a single-arm trial demonstrated that response rate was 40% and mPFS was 7 months, and OS was 12.7 months.^99^ A similar single-arm phase II trial (NCT00356889) of bevacizumab in combination with erlotinib but no traditional cytotoxic drugs in patients with unresectable BTC demonstrated a response rate of 18.4%, mOS of 9.9 months, and time to progression (TTP) of 4.4 months.^100^ In addition, atrial of multicenter phase II study (NCT01007552) of bevacizumab in combination with GC in advanced BTCs reported a 24% PR, 8.1 months of median PFS (mPFS), and 10.2 months of mOS.^101^A phase II study (NCT02053376) suggested promising efficacy of regorafenib (inhibitor of VEGFR1-3) in chemotherapy-refractory advanced/metastatic BTC, which demonstrated that mPFS was 15.6 weeks, mOS was 31.8 weeks, PR was 11%, and stable disease was 44% with a disease control rate of 56%. During the courses of these clinical trials, adverse reactions included hypertension (23%), hyperbilirubinemia (26%), hypophosphatemia (40%), and hand–foot skin reaction (7%).^102^ Some of trial failure events were also documented. Sorafenib, a multi-kinase inhibitor of VEGFR2/3, B-Raf, PDGFR-β, and C-Raf, showed a minimal level of drug efficacy in advanced BTC in a non-randomized phase II clinical trial with an ORR of 2%, the rate of stable disease at 12 weeks of 32.6%, PFS of 2.3 months (range: 0–12 months), and a mOS of 4.4 months (range: 0–22 months).^103^ Agreeing with this study, a multicenter, multinational phase II study (NCT01082809) revealed that sunitinib (inhibitor of multiple RTKs including VEGFR) monotherapy showed marginal efficacy in metastatic BTC patients. The median TTP was 1.7 months, the ORR was 8.9%, and the disease control rate was 50.0%.^104^ VEGFR2 antagonist vandetanib monotherapy or chemo combinations did not yield noticeable benefits in PFS in advanced BTC in a phase II trial (NCT00753675).^105^ Furthermore, a phase I trial (NCT02443324) of ramucirumab, a fully humanized monoclonal VEGFR2-targeted IgG antibody, reported that OR rate was 4%, and mPFS and mOS were 1.6 months and 6.4 months, respectively, in advanced BTC.^106^ A similar new phase II study of ramucirumab in patients with advanced BTC is presently ongoing (NCT02520141). Although the discrepancy from these individual clinical studies on angiogenic blockade remains to be mechanistically deciphered, a number of potential factors may be taken into account. First, monoclonal antibodies (e.g., bevacizumab) usually have higher specificity to bind to single proteins compared to small-molecule inhibitors that display a broader binding ability to block multiple proteins/kinases, likely eliciting off-target reactions. Second, some of the individual drug resistance cannot be neglected, as acquired drug resistance may rapidly develop in some patients who were not timely evaluated for the dynamic alterations of targeted receptors/molecules expression during the therapy. In addition, the divergent expression levels of VEGF/VEGFR and/or polymorphisms in GBC should be evaluated with respect to susceptibility to specific blockers. Finally, substantially longitudinal, case–control studies with large cohorts may be essential to establish the benefits of anti-angiogenic blockers. Nevertheless, at present, the combination regimen with angiogenic-targeted antibodies and other chemotherapeutic agents offers the optimal means to treat GBC.

### EGFR

EGFR, also commonly known as ErbB1 or HER1, was discovered to be associated with cancer development when v-ErbB oncogene of the avian erythroblastosis virus was observed in transformed chicken cells.^107^ Like HER2, EGFR activation triggers multiple intracellular downstream signaling cascades, including ERK/MAPK, PI3K-AKT, SRC, PLC-γ1-PKC, JNK, and JAK-STAT pathways,^108,109^ thus mediating cancer proliferation, angiogenesis, cell motility, adhesion, and metastasis.^110,111^ Elevated expression levels of EGFR were identified in non-small cell lung cancer (NSCLC) and malignant gliomas.^112,113^ In addition, constitutively activated mutations of EGFR existed in multiple cancers,^114,115^ thereby EGFR serves as a diagnostic and prognostic cancer biomarker, and also a potential target for cancer treatment.^116–118^

Identical to the earlier description regarding VEGF-targeted drugs, drugs that were developed to target EGFR mainly involve humanized monoclonal antibodies against the EGFR extracellular domain and small molecules of tyrosine kinase inhibitors (TKIs). Typical antibodies are cetuximab and panitumumab that are able to prevent EGFR from activated dimerization, thus inhibiting the downstream signaling.^119,120^ TKIs primarily include gefitinib, erlotinib, and afatinib, which have the ability to bind to the ATP-binding pockets on the intracellular catalytic kinase domain of RTKs, committing to the disruption of downstream signaling.^121^ To date, three generations of EGFR-TKI drugs have been evolutionarily devised to fight against mutation activity of EGFR.^122–124^ Erlotinib and gefitinib, representing the first generation of these TKIs, have the ability to compete reversibly with ATP binding at the tyrosine kinase domain of EGFR. As point mutation of EGFR T790M developed during the treatment of NSCLC patients with the first-generation EGFR TKIs,^125–127^ drug resistance emerged. To improve the efficacy, the second-generation EGFR TKIs such as afatinib and dacomitinib were created, which irreversibly inhibit ATP binding at the tyrosine kinase domain.^128^ In some of the clinical trials with afatinib, the drug failed to reach a level by which it effectively abolishes activity of T790M mutant EGFR.^129,130^ Subsequently, the third-generation TKIs osimertinib and olmutinib were formulated.^124^ Both drugs exhibited robust inhibition on the mutation activities of EGFR, as a favorable responses to osimertinib and olmutinib were achieved in 50–60% of patients with the T790M mutations.^131,132^ Thus, these two drugs have been approved as the second-line treatment drugs of patients who resist to the first-generation EGFR TKIs.

The overexpression population of EGFR in GBC was observed between 44 and 77% of patients in different independent studies.^133–135^ Elevated expression of EGFR in GBC tissues was positively correlated with poor prognosis of the patients.^136^ We also found that somatic mutations of EGFR were quite low ranging from 2.5 to 3.9% in GBC patients.^58,59^

A variety of therapeutic trials targeting EGFR in GBC patients have been completed, but endpoints were varied. Mody et al.^137^ reported a metastatic GBC case who received a combination treatment with gemcitabine (1000 mg/m^2^) on days 1 and 8 every 21 days and daily erlotinib (100 mg). The disease remained for 18 months with no progression after 12 cycles of combination therapy followed by maintenance with only erlotinib for 6 months. This result indicated the potential effective responses of EGFR-TKI on GBC therapy. Philip et al.^138^ reported a phase II study of erlotinib in 42 patients with advanced biliary cancer, in which 16 cases were GBCs. The overall confirmed response rate was 8% (3 patients; 95% CI 2–20) and the median TTP was 2.6 months (95% CI 2–4 months), while EGFR level was not associated significantly with clinical outcome. Lubner et al.^100^ published results of a multicenter phase II trial testing a combination of bevacizumab with erlotinib in 53 patients with unresectable biliary cancer (ClinicalTrial.gov NCT00356889). Twelve percent of patients (95% CI 6–27) had a confirmed PR. mOS was 9.9 months and TTP was 4.4 months. El-Khoueiry et al.^139^ reported ineffective outcomes in the phase II SWOG study on sorafenib and erlotinib in patients with 14 advanced GBCs and 20 cholangiocarcinomas. The combination of the two drugs resulted in two unconfirmed PRs (6%, 95% CI 1–20) with a mPFS of 2 months (95% CI 2–3), and a mOS of 6 months (95% CI 3–8 months) in the single-arm study. Cai et al.^140^ employed a meta-analysis to evaluate a combination therapy of EGFR-targeted drugs (erlotinib, cetuximab, or panitumumab) with GEMOX (gemcitabine and oxaliplatin) in 612 BTCs. The combination of GEMOX and EGFR-targeted therapy demonstrated improved PFS (HR 0.80, 95% CI 0.66–0.94, *P* = 0.03) compared with GEMOX alone, although OS was not significantly different. They also found that cholangiocarcinoma exhibited significantly greater benefits with 44% reduction from targeted therapy than non-cholangiocarcinoma including GBC and ampulla of Vater carcinomas that showed only 6% reduction in cholangiocarcinoma risk. Nonetheless, more clinical settings with combined regimens targeting EGFR are essential to offer a powerful tool for clinical practice.

### MAPK (RAS/RAF /MEK/ERK) pathway

MAPK signaling pathway is a crucial intracellular signal transduction that regulates varied cellular activities and is frequently dysfunctional in cancer.^141^ RAS/RAF/MEK/ERK is the most common pathway in MAPK signaling by which a variety of cancerous cells promote cell proliferation, death, differential, cell cycle progression, apoptosis, survival, metastasis, metabolism, and angiogenesis.^142–144^ A number of membrane receptors and intracellular proteins are directly or indirectly able to activate RAS, which includes K‐Ras, H‐Ras, and N‐Ras family members.^145^ Once the RAS protein is activated, it recruits RAF kinase family members such as Araf, Braf, or Craf to the plasma membrane and elicits downstream MEK.^146,147^ MEK has dual-specific serine/threonine and tyrosine kinase activity and share 80% sequence homology in MEK1 and MEK2.^148^ Activated MEK1/2 by RAFs induce phosphorylation of ERK1 or ERK2; then, pERK1 or pERK2 dimerizes and translocates to the nucleus, where it activates transcription factors to regulate gene expression.^149,150^ Approximately 40% of all human cancers involve altered MAPK pathway, including mutations of BRAF (~10%) and RAS (~30%).^151^ In GBC, KRAS point mutations were detected between 0 and 41% and BRAF gene amplifications existed in 5% patients.^152–157^

There was ample convincing evidence demonstrating that KRAS mutations mediate carcinogenesis of BTC by multiple research groups.^156,158–160^ Gln25His polymorphism of KRAS gene was identified to connect with GBC pathogenesis.^161^ Consistent with this result, KRAS rs61764370 polymorphism was intimately associated with risk and prognosis of cancers in 307 healthy controls and 541 GBCs in Indians.^162^ With regard to BRAF mutation frequency in GBC, it is still inconsistent, as no BRAF mutations were reported in the United States and Chile patients, but 33% of patients with BRAF mutations were present in Europe.^154,158^ However, the role of MEK/ERK in GBC has been relatively confirmed. MEK1/2 inhibitor trametinib inhibited GBC cells’ proliferation, migration, and invasion in a dose- and time-dependent manner, and induced GBC cell apoptosis in vivo and in vitro.^163^ Likewise, Horiuchi et al.^164^ demonstrated that MEK inhibitor U0126 abolished tumor liver invasion and increased survival of nude mice bearing human GBC cells.^16^ In addition, some traditional Chinese medicines, such as bufalin, pachymic acid, and artemisinin, had the ability to block GBC cell proliferation and invasion via interrupting MEK/ERK signaling.^165,166^ MiR-663a impaired MAPK/ERK pathway via altered regulation of EMP3 to suppress GBC progression.^167^ In contrast, lncRNA MALAT1, SLC25A22, and miR-101 augmented GBC cell proliferation through activating the MAPK/ERK pathway, leading to metastasis.^168–170^

A number of divergent therapeutic approaches in treatment with MAPK pathway blockers have been employed to unveil the potential targets for GBC patients. Giannini et al.^171^ reported that two cases yielded better therapeutic efficacy with PFS6months after receiving BRAF/MEK inhibitors treated for secondary GBC. Consistent with the report, Yu et al.^172^ also published an advanced secondary GBC case with a successful combination therapy with BRAF and MEK inhibitors after surgical excision, as there was no evidence of metastasis with PFS for 14 months and OS for 26 months after 8 months of treatment. Interestingly, a phase II study of GEMOX in combination with EGFR inhibitor cetuximab declared that KRAS mutations did not affect the difference in ORR and PFS between GEMOX and combination with EGFR inhibitor (NCT01308840, NCT01389414) in GBC,^173,174^ suggesting that the addition of cetuximab to gemcitabine and oxaliplatin did not seem to enhance the activity of chemotherapy in patients with GBC. In a phase II study of trametinib (GSK1120212, JTP-74057), the first generation of MEK1/2 inhibitor approved by Food and Drug Administration,^175^ trametinib showed safer and more effective drug responses than single gemcitabine treatment. Trametinib led to 10.6 months of PFS (95% CI 4.6–12.1), 20.0% of 1-year OS, 65% of stable disease, and 35% of PD in 20 Japanese patients with advanced BTC refractory to gemcitabine‐based therapy (NCT01943864).^176^ Similar results were obtained in SWOG S1310 study that recruited 44 patients (32% GBC patients) for trametinib treatment. The ORR of trametinib therapy was 10% (95% CI 0–23) vs. 8% (95% CI 0–19) seen in fluoropyrimidine therapy and the mPFS in trametinib therapy was 3.3 months in contrast to 1.4 months in fluoropyrimidine therapy.^177^ Some drug candidates with notable on-target adverse and toxicities events were limited to extensive clinical trials, including CI-1040 and PD0325901.^178,179^ Selumetinib (AZD6244, ARRY-142886), a second generation of MEK1/2 drug, was developed for the selective and uncompetitive small-molecule inhibitor of MEK1/2.^180^ A recent multi-institutional phase II study (NCT00553332) of selumetinib demonstrated that the drug response is of acceptable tolerability in patients with metastatic BTC, and that mPFS and mOS in selumetinib-treated cases were 3.7 months (95% CI 3.5–4.9 months) and 9.8 months (95% CI 5.97–not available), respectively.^181^

### PI3K/AKT/mTOR pathway

PI3K/AKT/mTOR signaling pathway is well known to participate in various biological and physiological cellular processes, including cell growth, mobility, differentiation, metabolic activity, and apoptosis.^182–184^ A wealth of oncogenic research evidence has established the notion that PI3K/AKT/mTOR pathway is one of the most key signaling pathways remarkably upregulated in a broad spectrum of cancers that involve breast cancer,^185^ NSCLC,^186^ gastric cancer,^187^ hepatocellular carcinoma,^188^ colorectal cancer,^189^ pancreatic cancer,^190^ cholangiocarcinoma,^191^ and GBC.^192^ Transmembrane growth factor receptors VEGFR, EGFR, insulin growth factor receptor 1, G protein-coupled receptors, and RAS proteins are capable of activating PI3K that phosphorylates phosphatidylinositol-4,5-bisphosphate (PIP2) to generate phosphatidylinositol-3,4,5-trisphosphate (PIP3). PIP3 subsequently binds to phosphoinositide-dependent kinase 1 (PDK1), thus phosphorylating and activating the serine/threonine kinase AKT.^193,194^ PTEN (phosphatase and tensin homolog), a tumor suppressor, is a phosphatase that dephosphorylates PIP3 into inactive PIP2, thereby dampening AKT and PDK1.^195,196^ Activated AKT can phosphorylate and activate mTOR or indirectly promote mTOR activity by phosphorylation and inactivation of tuberous sclerosis complex 1/2, a mTOR inhibitor. mTOR exists in two distinct complexes: mTOR complex 1 (mTORC1), which is composed of mTOR, Raptor, mLST8, and PRAS40; mTOR complex 2 (mTORC2) that consist of mTOR, Rictor, Sin1, and mLST8. mTORC1 activates S6 kinase 1 (also known as p70SK6) and promotes dissociation of eukaryotic initiation factor 4E binding protein 1 from eIF4E, stimulating cell growth and protein synthesis.^197,198^ As relatively limited understanding is available of mTORC1, the mTORC2 complex, insensitive to rapamycin, regulates actin cytoskeleton activity and controls AKT phosphorylation at Ser 473.^199,200^

Like other types of cancers that revealed important pathological impacts of the PI3K/AKT/mTOR pathway in cancer growth and metastases, mechanistic insights of GBC pathogenesis have advanced our knowledge that this pathway predominantly contributes to the initiation and progression of GBC. Lunardi et al.^201^ found that 90% of *Pten*^*+/−*^ mice with a high level of phosphorylated AKT developed GBC, highlighting an active role of PI3K/AKT signaling in the transformation of gallbladder epithelial cells. Leal et al.^202^ demonstrated that phospho-mTOR was positive in 64.1% of GBC patients and in 24% of chronic cholecystitis cases and that a high phospho-mTOR level in immunohistochemical analyses predicted poorer prognosis in patients with advanced GBC. PI3KCA mutations in GBC were differently reported in individual studies in the world. While no PIK3CA mutations were found in Brazil patients, 12.5%, 16.9%, and 21.4% of GBC with PIK3CA mutations were identified in USA, Japan, and Chile, respectively.^154,203–205^ We previously found that PIK3CA mutations E545K occurred in ~5.9% of GBC, and that these patients exhibited a worse prognosis.^58,206^ Epigenetic alteration of PTEN also contributes to the development of GBC as 30% GBCs exhibited PTEN promoter hypermethylated.^207^

Accumulating research evidence, not limited to PI3K, AKT, and mTOR, has also unveiled tumor-promoting function of important molecules that manipulate activation of the PI3K/AKT/mTOR pathway. For instance, M2 macrophages secrete CCL18 to promote GBC cell migration and invasion via activating PI3K/AKT pathway.^208^ EIF3D stabilizes GRK2 kinase that activates PI3K/AKT signaling pathway, rendering GBC cells invasive.^209^ Exploiting the same pathway, Nectin-4 and STYK1 promote GBC cell proliferation, metastasis, and tumor growth.^210,211^ In addition, ubiquitin protein ligase E3 component N-recognin 5 decreases the degradation of PTEN/PI3K/AKT signal pathway, facilitating tumor growth.^212^ Given the growing evidence that lncRNAs emerge to regulate gene expression, a number of laboratories have paid considerable attention in the identification of novel lncRNAs that prompt GBC progression. We recently discovered that lncRNA-HGBC stabilized by HuR promotes GBC cell proliferation, migration, and invasion by regulating miR-502-3p/SET/AKT axis.^32^ Analogous to our findings, PABPC1-stabilized lncRNA-PAGBC acted as a miRNA sponge to activate the PI3K/AKT/mTOR pathway, committing to tumor growth and metastasis.^33^ Specificity protein 1-induced lncRNA LINC00152 up-regulates PI3K/AKT pathway and contributes to GBC cell growth and tumor metastasis.^213^ Jin et al.^214^ demonstrated that miR-143-3p targets ITGA6 to inhibit PI3K/AKT pathway, thus suppressing GBC growth and angiogenesis. Overall, the identification of a great number of key factors has pointed to novel targets potential for therapy in GBC. Several inhibitors targeting the PI3K/AKT/mTOR pathway, including A66, Wortmannin, and LY294002, have been demonstrated to inhibit GBC cell proliferation, migration, and invasion both in vitro and in vivo.^215–217^ In addition, rapamycin, RAD001, and AZD8055 reported by Leal et al.^218^ were able to block mTOR and inhibit the growth and migration of GBC cells in vitro. In a transgenic mouse model, rapamycin can also inhibit the incidence of GBC.^219^ OSI-027 blocked mTOR, enhancing the sensitivity of GBC cells to 5-fluorouracil.^220^ Some of the traditional Chinese medicines, such as bufalin, liensinine, and dioscin, also received striking attention capable of inhibiting GBC cell proliferation and inducing cell apoptosis via targeting the PI3K/AKT pathway.^166,221,222^

In the clinic, a phase I trial (NCT00949949) was performed to determine the maximum tolerated dose (MTD) of mTOR inhibitor everolimus (5 mg) combined with either gemcitabine (800 mg/m^2^, Cohort I) or gemcitabine plus cisplatin (12.5 mg/m^2^, Cohort II) in cancers. In this clinical setting, 10 patients with cholangiocarcinoma or GBCs were abstracted for the Cohort III trial treated with three drugs. The results showed that six patients remained stable, whereas four developed a progressive stage, suggesting that the three-drug combination course may offer favorable benefit to GBC.^223^ A multi-institutional phase II study of MK-2206 (NCT01425879) demonstrated that MK-2206, a single-agent targeting AKT, exhibited acceptable tolerability in eight patients with advanced, refractory BC.^224^ Regarding the multiple drug candidates available from the preclinical trials described early, it is worthwhile interrogating if some of these agents function to block the PI3K/AKT/mTOR pathway in GBC patient, thereof offering additional opportunities for patient treatment.^225^

### PD-1/PD-L1

**Fig. 3:**
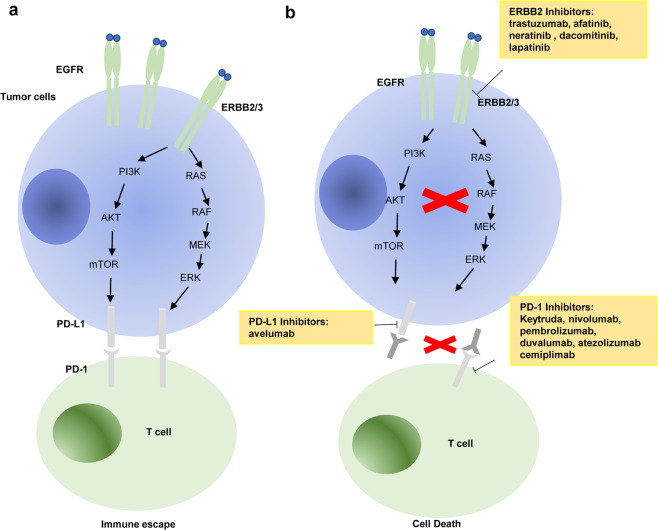
Immunoregulation of PD-L1/PD-1 in GBC. **a** Activated ERBB2/ERBB3 mutations upregulate PD-L1 expression through activation of the PI3K/AKT signaling and RAS/RAF/MEK/ERK pathway to induce immune evasion of GBC cells. **b** Yellow boxes highlight that drug targeted ERBB2, PD-1, or PD-L1 could dampen immune evasion of GBC cells

In the recent few years, intense research evidence focusing on antitumor immunity has established the proof of concept that blockade of the interaction between tumor-derived checkpoint ligands and their corresponding binding receptors expressed by T cells elicits T cell immunity against tumors.^106,226^ PD-L1, also known as B7-H1 or CD274, is a membrane-associated protein and specifically binds to PD-1 expressed on T cells.^227^ Interaction of these immune checkpoint proteins leads to disruption of major histocompatibility complex coupled with T cell receptors, the identical interaction of membrane proteins by which cytotoxic T cells recognize and eliminate tumor cells (Fig. 3). While the aberrant expression of PD-L1 in multiple cancers enables tumor cells to escape the host immune surveillance and drive tumor metastasis, little is known regarding mechanistic regulation of PD-L1 and PD-1 underlying GBC development. We are the first group, to our knowledge, to report that ectopic expression of ErbB2/ErbB3 mutants in GBC cells upregulated PD-L1 expression, which suppressed T cell-mediated cytotoxicity and drove tumor growth and metastasis.^228^ Mechanistically, ErbB2/ErbB3-mediated expression of PD-L1 was dependent on activation of the PI3K/Akt signaling pathway. The results demonstrated that acquired ERBB2/ERBB3 mutations by tumor cells are essential to induce checkpoint PD-L1, rendering tumor cells evasive from cytotoxic T cell immunity against tumor. Agreeing with our findings, Gong et al.^229^ found that PLAC8 increased PD-L1 expression and conferred tumor resistance to gemcitabine and oxaliplatin, providing an alternative opportunity with PD-1/PD-L1 blockers to treat chemotherapy-refractory patients with GBC.

There were accumulating literature reports analyzing the relationship between expression levels of PD-L1 and/or PD-1 and clinical outcomes. Elevated PD-L1/PD-1 levels were correlated with poor survival of cancer patients with NSCLC,^230^ melanoma,^231^ gastric cancer,^232^ adrenocortical carcinoma,^233^ breast cancer,^234^ hepatic cancer,^235^ and pancreatic cancer.^236,237^ Specifically, PD‐L1 expression in GBC (23%) was comparable to breast cancer (23%), urothelial cancer (20%), and pulmonary squamous cell carcinoma (27%).^238,239^ Mody et al.^240^ found that 12% patients with tumor cell-expressing PD-L1 and 55% patients with tumor-infiltrating lymphocyte (TIL)-expressing PD-1 in a total of 203 GBC patients were associated with corresponding genetic aberrations and tumor mutational burden (TMB) status. Likewise, Lin et al.^241^ showed that 18% of 66 GBC patients were positive for PD-L1 expressed by tumor cells. Ha et al.^242^ measured the serum level of the soluble form of PD-L1 (sPD-L1) in 158 advanced BTC patients, and found that patients with high sPD-L1 (≥0.94 ng/mL) showed decreased OS than patients with low sPD-L1 (7.93 vs. 14.10 months, *p* < 0.001). Kim et al.^243^ observed that the mOS of low and high PD-L1 expression of 101 primary GBC cases was 50.13 ± 3.14 and 27.88 ± 6.69 months (*P* value = 0.049), respectively, and the mPFS was 49.48 ± 3.29 and 23.33 ± 7.47 months (*P* value = 0.028), respectively. These individual studies underscore that PD-L1 serves as an independent marker for the prognosis of GBC. Not surprisingly, there was a limited observation that did not fully support these earlier findings. Neyaz et al.^239^ recruited 174 cases of GBC and found that mOS in PD‐L1‐negative (PD-L1^−^) and PD‐L1-positive (PD-L1^+^) cases were 12.0 and 14.0 months, respectively (*P* = 0.546). Another study showed that PD-1^+^ in TILs and PD-L1^+^ in tumor cells did not correlate with OS or PFS, but a high density of CD8^+^ TILs in PD-L1^−^ tumors was positively correlated with OS (*P* = 0.002) and PFS (*P* = 0.014), indicating that CD8^+^ TILs is an additional marker for disease prognosis, except PD-L1 and PD-1. Although we do not have sufficient knowledge to explain the inconsistency, the research focusing on tumor immune checkpoint proteins has opened a new era to explore their potential roles in mediating tumor ability to escape immune surveillance.

Treatment of GBC patients with PD-1/PD-L1 inhibitors has emerged as a promising strategy for targeted therapies (Table 2).^244,245^ TMB-H and MSI-H are potentially useful for assessing neoantigen presentation and viability of immune checkpoint inhibition.^246,247^ Weinberg et al.^248^ reported that PD-L1 overexpression was seen on GBC tumor cells from 19 of 237 (8.0%) tumors using IHC. Increased MSI-H and TMB-H were, respectively, seen in 1 out of 104 tumors (1.0%) and 6 out of 104 tumors (5.8%) using NGS. All of these results suggested that PD-L1 in GBCs is a therapeutic marker for immune checkpoint blockade (12%). In 2019, Kong et al.^249^ reported a successful immune therapy case that expressed strong PD-L1 expression (≥50%) and gave rise to substantial responses to nivolumab immunotherapy after the failure of multiple lines of therapies. Indeed, multiple clinical studies have demonstrated the efficacy of PD-L1/PD-1 inhibitors in the treatment of GBC. In a multicenter, case-controlled phase I trial named MakotoUeno (ID: JapicCTI-153098), 30 unresectable and recurrent BTC patients were enrolled into two cohort trials: patients received nivolumab only (240 mg every 2 weeks) as the monotherapy; patients received nivolumab (240 mg every 2 weeks) and cisplatin (25 mg/m^2^) plus gemcitabine (1000 mg/m^2^) as combined regimens.^250^ The results showed that the mOS was 5.2 months (90% CI 4.5–8.7) vs. 15.4 months (90% CI 11.8–not estimable), and mPFS was 1.4 months (90% CI 1.4–1.4) vs. 4.2 months (90% CI 2.8–5.6), respectively, for the first and second cohort trials. In a single-arm phase II study of nivolumab in BTC patients (NCT02829918), the mPFS was 3.68 months (95% CI: 2.33–5.98) and the mOS was 14.24 months (95% CI 6.64–NA).^251^ In addition, 6- and 12-month OS was obtained in 71.4% and 52.3% populations and 6- and 12-month PFS was seen in 35.2% and 24.1%, respectively. These results indicated that nivolumab is a manageable and effective agent for BTC patients. In the KEYNOTE-028 (NCT02054806; phase I) and KEYNOTE-158 (NCT02628067; phase II) study, BTC patients received pembrolizumab treatment.^252,253^ In KEYNOTE-028 study, objective response rate (ORR) was 13.0% (3/23, all PR; 95% CI 2.8‒33.6) and median duration of response (DOR) was not reached (NR; range 21.5–29.4+ month). mOS and mPFS were 6.2 months (95% CI 3.8‒10.3) and 1.8 months (95% CI 1.4‒3.7), respectively. Furthermore, ORR of PD‐L1^+^ patients (*n* = 61) and PD‐L1^−^ patients (*n* = 34) was 6.6% (4/61) and 2.9% (1/34), respectively. In KEYNOTE-158, ORR was 5.8% (6/104, all PR; 95% CI 2.1–12.1) and median DOR was NR (range 6.2–23.2+ months). mOS and mPFS were 7.4 months (95% CI 5.5–9.6) and 2.0 months (95% CI 1.9–2.1), respectively. The results showed that pembrolizumab provides durable antitumor activity, regardless of PD-L1 expression. Unlike KEYNOTE‐028 study, the trail (NCT02443324) recruited both PD-L1^+^ and PD‐L1^−^ patients, in which 46.2% of PD-L1 patients underwent tumor recurrence or metastasis. ORR was 4%. mOS and mPFS were 6.4 and 1.6 months, respectively. PD‐L1^+^ patients had improved OS compared with PD‐L1^−^ cases (11.3 vs. 6.1 months), but there is no difference in mPFS (1.5 vs. 1.6 months).^106^ All the results suggest that PD-1/PD-L1 inhibitors are safe and effective in treating GBC.

**Table 2:** PD-L1/PD-1 studies with reported outcome in GBC

Clinical trail number	Study phase	Treatment agent	Checkpoint target	Number of patients	Outcome	Ref.
NCT02443324	I	Pembrolizumab + ramucirumab	PD-1 + VEGFR	26	PR: 3.8%, mPFS: 1.64 months, mOS: 6.4 months	^106^
JapicCTI-153098	I	Nivolumab	PD-1	34 (33% GBC)	PR: 37%, mPFS: 4.2 months, mOS: 15.4 months	^250^
		Nivolumab with chemotherapy		30 (33% GBC patients)	PR: 3%, mPFS: 1.4 months, mOS: 5.2 months	
NCT02829918	II	Nivolumab	PD-1	54 (26% GBC)	PR: 22%, DCR: 60%, mPFS: 4 months, mOS: 14.2 months	^251^
NCT02054806	I	Pembrolizumab	PD-1	24 (membranous PD-L1 ≥1%)	PR: 13%, SD: 17%, mPFS: 1.8 months, mOS: 6.2 months	^252^
NCT02628067	II	Pembrolizumab	PD-1	104	PR: 5.8%, mPFS: 2 months, mOS: 7.4 months	^253^
NCT01938612	II	Duvalumab with/without tremelimumab	PD-L1	42 (45% GBC)	PR: 48%, mPFS: 1.5 months, mOS: 8.1 months	^260^
			PD-L1 + CTLA-4	65 (25% GBC)	PR: 11%, mPFS: 1.6 months, mOS: 10.1 months	
NCT01853618	I	Tremelimumab + RFA	PD-L1 + CTLA-4	20 (10% GBC)	PR: 12.5%, mPFS: 3.4 months, mOS: 6 months	^261^
NCT02699515	I	M7824	PD-L + TGF-β	30 (40% GBC)	ORR: 20%, mOS: 12.7 months	^262^

Combined regimens of anti-PD-L1/PD-1 checkpoint agents with other therapies have also been increasingly practiced in clinic, including additional immunotherapies, chemotherapy, and targeted therapies.^254–260^ In a phase I study led by Xie’s group (NCT01853618) showed that PFS and OS were 3.4 months (95% CI 2.5–5.2) and 6.0 months (95% CI 3.8–8.8),^261^ which declared that tremelimumab (anti-CTLA-4 monoclonal antibody (mAb)) is a potential treatment strategy for patients with advanced BTC. A phase I study (NCT01938612) evaluated durvalumab (anti-PD-L1 mAb) with/without tremelimumab in Asian GBC patients. Median DOR was 9.7 and 8.5 months for single durvalumab and durvalumab + tremelimumab treatment, respectively. mOS was 8.1 (95% CI 5.6–10.1) months and 10.1 (95% CI 6.2–11.4) months for the single and dual treatment, respectively, suggesting that conjunction of anti-PD-L1/PD-1 with anti-CTLA-4 therapies may hold promising efficacy for patients with GBC. Thus, comprehensive trials utilizing anti-CTLA-4 antibody combined with anti-PD-L1/PD-1 antibodies in GBC are currently underway, as this approach yielded noticeable benefits to other types of cancer patients. Fujiwara et al.^262^ employed a new therapeutic tool engineering a bifunctional fusion protein that targets PD-L1 and transforming growth factor-β (M7824) to treat Asian patients with advanced solid tumors (including 40% GBC patients) (NCT02699515). In this trial, three patients discontinued M7824 treatment due to treatment-related adverse events (the cases of bullous pemphigoid, colitis, and gastroparesis). This endogenous interruption targeting both molecules needs to be substantially evaluated in future. While multiple clinic settings with immune checkpoint therapies combined with other therapies wait for outcomes at present, we are fully confident with this novel approach able to yield greater benefits to GBC patients, ultimately improving life quality.

### DNA damage repair (DDR) pathway

DDR can execute full repairing or elimination of damaged cells to protect host organisms against possible carcinogenesis.^263^ There are four major DDR pathways identified in the cells, for example, base excision repair, nucleotide excision repair (NER), double-strand break repair and mismatch repair (MMR).^264^ As the intracellular events participate in the pathogenesis of GBC, DDR pathways act an active role to contribute to the development of this disease. Fang et al.^265^ reported that a dual-specific phosphatase DUSP1 enhances the chemoresistance of GBC via the modulation of the p38 pathway and DNA damage/repair system. Suppression of cholesterol biosynthesis by lovastatin could inhibit GBC cell proliferation, possibly through attenuating the DDR process.^266^

Abdel-Wahab et al.^267^ have clustered 20 “direct” DDR genes (*ATM*, *ATR*, *BRCA1*, *BRCA2*, *FANCA*, *FANCD2*, *MLH1*, *MSH2*, *MSH6*, *PALB2*, *POLD1*, *POLE*, *PRKDC*, *RAD50*, *SLX4*) and “caretaker” genes that regulate DDR process (*BAP1*, *CDK12*, *MLL3*, *TP53*, *BLM*) in BTC (270 ICC, 60 ECC, and 92 GBC specimens) by hybrid capture-based NGS. BRCA-associated BTC is uncommon as BRCA1/2 mutations were detected in 4.0% of 353 GBC samples by Spizzo. However, they found a correlation of BRCA-mutant BTC with MSI-H/dMMR, which represented an additional predictive marker for response to checkpoint inhibition.^268^ Consistent with these reports, Javle et al.^269^ identified 7.8% BRCA2 or ATM mutations in 623 advanced GBC patients.

The alterations of DDR genes increase the sensitivity of anti-cancer chemotherapy and radiation treatments. The recent researches suggest that specific DDR gene mutation or expression may have an impact on response to platinum-based chemotherapy in patients diagnosed with BTC.^270^ Hwang et al.^271^ evaluated the effect of ERCC1 on treatment outcomes in advanced BTC patients treated with platinum-based chemotherapy, which is the 5′ endonuclease of the NER complex to prevent damage to DNA by NER. They found that mPFS and mOS were significantly longer in ERCC1^−^ group than in ERCC1^+^ group of cisplatin-treated group (4.6 vs. 1.9 months, *P* = 0.014; 9.1 vs. 7.9 months, *P* = 0.017). Baek-Yeol Ryoo et al.^272^ revealed that DDR gene mutations were found in 62.5% of BTC patients (including 20.2% GBC patients), and that DDR gene mutations associated with longer mPFS (6.9 vs. 5.7 months; *P* = 0.013) and mOS (21.0 vs. 13.3 months; *P* = 0.009) in patients with BTC treated with first-line platinum-based chemotherapy for unresectable or metastatic disease. These results indicate that mutations in DDR genes may serve as predictive biomarkers for the response to platinum-based chemotherapy in patients with BTC.

To date, clinical trials clarifying the efficacy of DDR inhibitors in GBC patients have not been reported, but poly (ADP-ribose) polymerase (PARP) inhibitors have been developed in monotherapy that is engaged for patients with HR deficiency (i.e., BRCA mutation) and also in BRCA-like tumors. At present, a clinical trial (NCT03878095) targeting PARP is waiting for final results.

### C-mesenchymal–epithelial transition factor (MET)

MET is an oncogene encoding tyrosine kinase receptor of the hepatocyte growth factor (HGF). Once HGF binds to MET, the receptor undergoes dimerization and induces downstream signaling pathways, such as PI3K/AKT, RAS/RAF/MEK/ERK, and Wnt/β-catenin signaling,^273–275^ which regulate cell proliferation, metastasis, and drug resistance. Elevated MET was associated with poor prognosis in liver cancer, breast cancer, pancreatic cancer, gastric cancer, NSCLC, cervical cancer, and colorectal cancer.^79,276–285^ In GBC, MET overexpression ranged from 5 to 74% of patients, and was also associated with clinical poor outcome.^286–290^ The conclusion was further supported by different groups that found a similar correlation.^288^ In addition, NK4, an HGF inhibitor, inhibited tumor growth and invasion of GBC in animal models.^291–294^ Inconsistent with these findings, Kim et al.^295^ reported dissimilar results that no such correlation between MET expression and poor prognosis was significantly obtained. Up to date, there are three categories of MET inhibitors available in the clinic: small molecules targeting MET receptors (e.g., crizotinib, tivantinib, savolitinib, tepotinib, cabozantinib, and foretinib), MET receptor mAbs (e.g., onartuzumab), and antibodies against its ligand HGF (e.g., ficlatuzumab and rilotumumab).^296–299^ A clinical trial (NCT03027284) is currently settled to evaluate the feasibility and efficacy of MET inhibitors in the treatment of GBC.

### TP53

TP53 is an important tumor suppressor gene and its mutations are commonly detected in 50% of almost all human cancers, such as colon cancer, pancreatic cancer, breast cancer, and hepatobiliary cancer.TP53 is known to participate in the cellular DNA damage response, and induction of cell cycle arrest and apoptosis.^300,301^ Magnolol, an organic compound derived from Chinese traditional medicine, was found to upregulate P53, resulting in interrupting cell cycle progression at theG0/G1 phase and inducing mitochondrial-mediated apoptosis. However, this cell death effect was prevented by pretreatment with a p53 inhibitor pifithrin-a.^302^ Tian et al.^303^ also demonstrated that apoptosis-stimulating of p53 protein 2 activated p53 and recruited macrophages via PKC-ɩ/GLI1 pathway, committing to inhibition of metastasis.

It is of note that the expression of p53 in GBC is ethnically related to different race populations. The TP53 mutations in Greek GBC patients were lower than those in Japan and Chile GBC patients. One-third of the north Indian patients with GBC have mutations in exons 5–8 of p53 gene.^304^ Moreover, different types of TP53 mutations in GBC were defined in Japan, Chile, and Hungary.^159,305,306^ It is quite interesting to mechanistically understand the genetic variations associated with geographic difference.

Overexpression and high mutation frequency of p53 protein, which has tumor-promoting signature rather than cell apoptotic activity, were correlated with a poor survival of a broad type of cancers, including GBC,^307–309^ thus serving as a cancer biomarker. We previously analyzed GBC mutagenesis and found that TP53 was ranked as the top one in a large spectrum of mutated genes in GBC.^58,228^ In addition, Singh et al.^310^ demonstrated that p53 is an independent prognostic factor for the poor prognosis of GBC (*P* = 0.03; HR: 5.63; 95% CI 1.21–26.26). Supporting this notion, a variety of studies also pointed to the mutated p53 as a prognostic marker of GBC.^300,311^ P53 expression together with other factors was engaged to predict poorer prognosis of GBC, such as cyclin D1, Ki-67, p16, and MSH2.^300,307,312^ Interestingly, some divergent evidence was also documented in the literature. One hundred and three (44.8%) of 230 GBC cases expressed mutant p53 protein that was not correlated with clinical parameters such as tumor growth.^313^ Likewise, Hidalgo Grau et al.^314^ reported p53 protein nuclear overexpression in 41 GBCs was not associated with poor histological differentiation, gallbladder wall invasion, or patient survival. Although these distinct conclusions are still controversial, mechanistic insights should be in parallel taken into account, as a variety of p53 mutation forms together with multiple phosphorylation sites coexist, implicating dissimilar roles played by p53 in tumor development, such as tumor-promoting effects and loss of tumor suppressor activity.

Although TP53 has the highest mutation rate in GBC, very limited clinical trials targeting mutated p53 were reported to evaluate potential therapeutic benefit. Makower et al.^315^ led to a phase II clinical trial of oncolytic adenovirus ONYX-015(dl1520, CI-1042), which intervenes the p53 pathway in 19 patients with hepatobiliary tumors, in which 15 cases expressed p53 mutations, and 5 patients had GBCs.^316^ In this study, 16 patients responded to the viral product in intralesional treatment, while serious toxicities (>grade 2) were rarely observed. The comprehensive analyses of this approach for therapeutic efficacy and safety remain to be clarified.

### CDKN2A/B

CDKN2A/B, a cyclin-dependent kinase inhibitor 2A/B, inhibits CDK4 and CDK6, and also prevents pRB phosphorylation, thus leading to cell cycle arrest at the G1/S phase.^317–319^ In an analysis of the comprehensive genomic profiling with NGS, *CDKN2A/B* gene is one of the most frequently mutated genes in 108 Chinese and 107 US GBC patients. The altered rate with 26% in Chinese patients was identical to 25% seen in US populations.^320^ More interestingly, the coincidence of ERBB2 genetic mutations with CDKN2A/B variations in US patients was stronger (odds ratio 10.8, *P* = 0.0001) than those in Chinese cohort (odds ratio 5.4, *P* = 0.0014), which suggests that CDKN2A/B alterations were significantly associated with distant metastases.^321^ Our previous study showed that CDKN2A/B mutation rate was ~5.9% in GBC.^58,228^ There were other reports supporting the notion that CDKN2A/B mutations mediate the pathogenesis of GBC.^248,322^ Collectively, all the data indicate that CDKN2A/B is a potential target for GBC therapy. It was also noted that Leiting et al.^323^ found that CDKN2A was not associated with the survival of BTC. Nonetheless, to date, no potential drug candidates have been explored to target CDKN2A/B in cancer treatment.

### KIT

KIT is a type III transmembrane RTK expressed in a variety of human cells.^324^ The downstream signaling of KIT mainly involves MAPK, PI3K/AKT, and JAK/STAT pathways, thus participating in the regulation of cell proliferation, differentiation, growth, survival, and migration.^325–327^ Interestingly, the expression of KIT of patients with gallstones was lower in gallbladder tissue than that in healthy subjects.^328^ However, in GBC, the expression of KIT was elevated.^329^ At present, at least 16 KIT inhibitors are available for the blockade of its activity elevated in various types of cancers, including leukemia, prostate cancer, gastrointestinal stromal tumor, and renal carcinoma.^330–333^ Typical therapeutic blocking agents employed to target KIT include cabozantinib, dovitinib, masitinib, and pazopanib.^334–336^ At present, two additional clinical trials (NCT01153750, NCT02115542) wait for final results.

## Challenges and future perspective

**Fig. 4:**
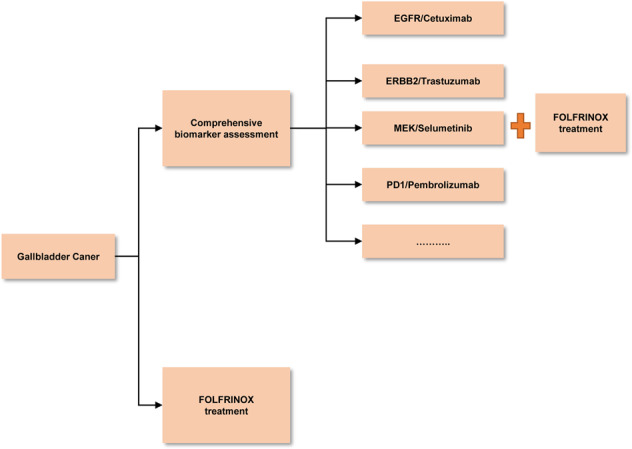
Schematic of the clinical trial in our center (NCT03768375). Based on genomic and proteomic profiling of participants, patients received personalized targeted drugs with FOLFRINOX treatment or just FOLFRINOX treatment

Given that GBC, a high-grade malignant tumor, is mostly of contraindication to surgical removal, great effort has been added to devise the therapeutic strategies and improve the efficacy of non-surgical treatment, including developing new specific agents and therapeutic means, such as targeted drugs, vaccines, and nanoparticles. We are particularly encouraged by the tremendous achievement in a number of recent clinical trials focusing on the efficacy of targeted drugs as described earlier in the text, since the favorable endpoints of these trials are at least, in part, attributed to the rapid development of the state-of-art measures, including NGS, human WES, transcriptomic (RNA array, RNAseq), proteomic, epigenetic, and metabolomic profiling that assist evaluation of genetic signature, drug action mechanisms, and therapeutic responses.^337^ Therefore, these clinical attempts are anticipated to be of great benefits for the treatment devises. It is concomitantly worthwhile to note that a number of unexpected obstacles coexist and even persist throughout the entire clinical trials, resulting in the elimination of a majority of drug candidates, terminating trials, or leading to testing failure. These typical unfavorable events involve apparent drug side effects or off-target toxicities particularly in the multiple chemotherapeutic drug applications, and decreased responses or no responses due to rapid development of resistance. In addition, other scenarios cannot be ignored, including individual genetic difference, tumor gene mutations, and tumor heterogeneity that otherwise coordinately bypass the targeted therapy. Hence, the therapeutic efficacy of promising drug candidates together with potentially adversary impacts should be warily taken into account. To achieve overall benefits, it is highly recommended to exploit combination targeted therapies aiming at different key pathways underpinning cancer metastasis, which expectedly yield synergistic efficacy with minimal toxicities. Of note, the newly created tumor models transplanted directly with patient-derived tumors such as PDX/PDTX and patient-derived organoids have offered a great opportunity for drug sensitivity and/or resistance screening, as it is of paramount importance for devising sensitive treatment in personalized medicine. In addition, it is also emerging that the tumor immune therapy has paved an exciting novel therapeutic avenue to improve the efficacy of GBC treatment. In context with recent findings of the key roles played by PD-L1 immune checkpoint and other signaling molecules in GBC, we have begun a multicenter clinical phase II trial (NCT03768375) employing an umbrella design, which aims to evaluate the efficacy of different courses of treatment based on genomic and proteomic profiling combined with FORFIRINOX in advanced or recurrent GBC patients (Fig. 4). We expect that these multiple combination targeted settings yield distinct levels of benefits to participants, thus offering great value for future clinical practice, particularly in personalized medicine. To this end, it is urgent to establish a fruitful platform to collaborate globally with divergent research institutions, laboratories, and hospitals, ultimately giving rise to an optimal course to fight against this fatal disease.
